# Taxonomic, Physiological, and Biochemical Characterization of *Asterarcys quadricellularis* AQYS21 as a Promising Sustainable Feedstock for Biofuels and ω-3 Fatty Acids

**DOI:** 10.3390/plants13213008

**Published:** 2024-10-28

**Authors:** Nam Seon Kang, Sung Min An, Chang Rak Jo, Hyunji Ki, Sun Young Kim, Hyeon Gyeong Jeong, Grace Choi, Ji Won Hong, Kichul Cho

**Affiliations:** 1 National Marine Biodiversity Institute of Korea, Seocheon 33662, Republic of Korea; kang3610@mabik.re.kr (N.S.K.); sman@mabik.re.kr (S.M.A.); happyccr@mabik.re.kr (C.R.J.); hki@mabik.re.kr (H.K.); sykim@mabik.re.kr (S.Y.K.); hgjeong@mabik.re.kr (H.G.J.); gchoi@mabik.re.kr (G.C.); 2 Department of Hydrogen and Renewable Energy, Kyungpook National University, Daegu 41566, Republic of Korea; jwhong@knu.ac.kr; 3 Advanced Bio-Resource Research Center, Kyungpook National University, Daegu 41566, Republic of Korea

**Keywords:** *Asterarcys quadricellularis*, morphological characterization, fatty acids, biofuels, microalgae, molecular identification

## Abstract

*Asterarcys quadricellularis* strain AQYS21, a green microalga isolated from the brackish waters near Manseong-ri Black Sand Beach in Korea, shows considerable potential as a source of bioactive compounds and biofuels. Therefore, this study analyzed the morphological, molecular, and biochemical characteristics of this strain; optimized its cultivation conditions; and evaluated its suitability for biodiesel production. Morphological analysis revealed characteristics typical of the *Asterarcys* genus: spherical to ellipsoidal cells with pyrenoid starch plates and mucilage-embedded coenobia. Additionally, features not previously reported in other *A. quadricellularis* strains were observed. These included young cells with meridional ribs and an asymmetric spindle-shaped form with one or two pointed ends. Molecular analysis using small-subunit rDNA and *tuf*A sequences confirmed the identification of the strain AQYS21. This strain showed robust growth across a wide temperature range, with optimal conditions at 24 °C and 88 µmol m^−2^s^−1^ photon flux density. It was particularly rich in ω-3 α-linolenic acid and palmitic acid. Furthermore, its biodiesel properties indicated its suitability for biodiesel formulations. The biomass of this microalga may serve as a viable feedstock for biodiesel production and a valuable source of ω-3 fatty acids. These findings reveal new morphological characteristics of *A. quadricellularis*, enhancing our understanding of the species.

## 1. Introduction

Microalgae are mostly single-celled eukaryotic organisms that thrive in diverse aquatic environments, including freshwater, oceans, and even extreme conditions [1,2]. They play key roles in the global carbon and nitrogen cycles, as they are crucial primary producers of these elements [3]. Moreover, their ability to convert atmospheric CO_2_ into valuable organic compounds through photosynthesis has attracted growing attention from various industries [4,5]. This interest is primarily due to their abundant reserves of lipids, proteins, carbohydrates, pigments, vitamins, and other compounds [6,7] with applications in bioenergy, nutraceuticals, pharmaceuticals, food, cosmetics, and agriculture [8,9,10,11].

Microalgae exhibit a photosynthetic efficiency and biomass productivity much higher than those of terrestrial plants [12]. In addition, they can survive in harsh conditions such as brackish water and wastewater [12,13]. Their sustainability and lack of competition with agricultural land make them a valuable resource [14,15]. Furthermore, the implementation of the Nagoya Protocol has spurred global efforts to secure biological resources, such as microalgae, which are essential for national competitiveness and biological resource sovereignty [16,17].

Chlorophyta, a major phylum within green algae, includes various species with extensive geographic distribution across freshwater, marine, and terrestrial environments [18,19,20,21]. These microalgae maintain the global ecological balance in aquatic environments [22,23]. Biomass from Chlorophyta species is a vital resource in biotechnology [24,25]. Despite this significant role in marine ecosystems and biotechnology, the classification of these microalgae has not been comprehensively documented [26,27].

The complexity of obtaining detailed morphological data through electron microscopy and the lack of comprehensive DNA sequencing has often led to misidentification that underestimates the ecological significance and biotechnological potential of microalgae [26]. Accurate species identification and classification ensure the safety of microalgal products and protect public health [26,28,29,30]. Therefore, isolating and establishing clonal cultures from various environments is essential for precise species identification that effectively elucidates their commercial value and ecological roles.

The genus *Asterarcys*, described by Comas Gonzalez [31], includes only one officially recognized species, *Asterarcys quadricellularis* [32]. The genus is a potential producer of biofuels [33], in addition to producing unsaturated fatty acids [34]. Furthermore, it generates valuable bioactive compounds such as carotenoids [35]. It is also effective in water purification and serves as an effective biofertilizer ingredient in edible crops, owing to its high protein content [36,37,38,39]. These attributes highlight its potential as a raw material across various industrial sectors and emphasize the need for further research to expand its applicability.

The genus *Asterarcys* is part of the phylum Chlorophyta. Although it includes only one recognized species, *A. quadricellularis*, it thrives in various environments, including freshwater [40] and terrestrial [34,38] ecosystems, demonstrating ecological adaptability within green microalgae. The adaptability of this genus emphasizes its essential role in the aquatic food chain and ecosystem productivity. However, its presence in saline or brackish environments is underreported. Thus, the detection of *Asterarcys* across diverse habitats is vital for understanding the biodiversity and ecological impact of this genus.

Microalgal strains can demonstrate a wide range of physiological activities and variations in metabolite profiles, even among individuals of the same species [41,42]. Despite the ecological and economic significance of *Asterarcys*, comprehensive taxonomic information on this genus is lacking, leading to an underestimation of its potential ecological and biotechnological applications. Therefore, accurate identification and the establishment of clonal cultures are crucial for advancing research and fully realizing the potential applicability of *Asterarcys* in both fields.

The objectives of the present study were (1) to comprehensively analyze the morphological and molecular characteristics of the *A. quadricellularis* strain AQYS21 isolated from brackish waters in Korea, (2) to investigate its optimal culture conditions, (3) to evaluate its potential as a sustainable source of biofuels and essential fatty acids, and (4) to assess its lipid, carbohydrate, and protein contents, along with its fatty acid composition and biodiesel properties. The findings from this study may enhance the taxonomic understanding of *A. quadricellularis* AQYS21 and facilitate its broad industrial application, particularly in the fields of biofuels, essential fatty acids, and nutraceuticals.

## 2. Results

### 2.1. Morphological Characteristics

**Figure 1:**
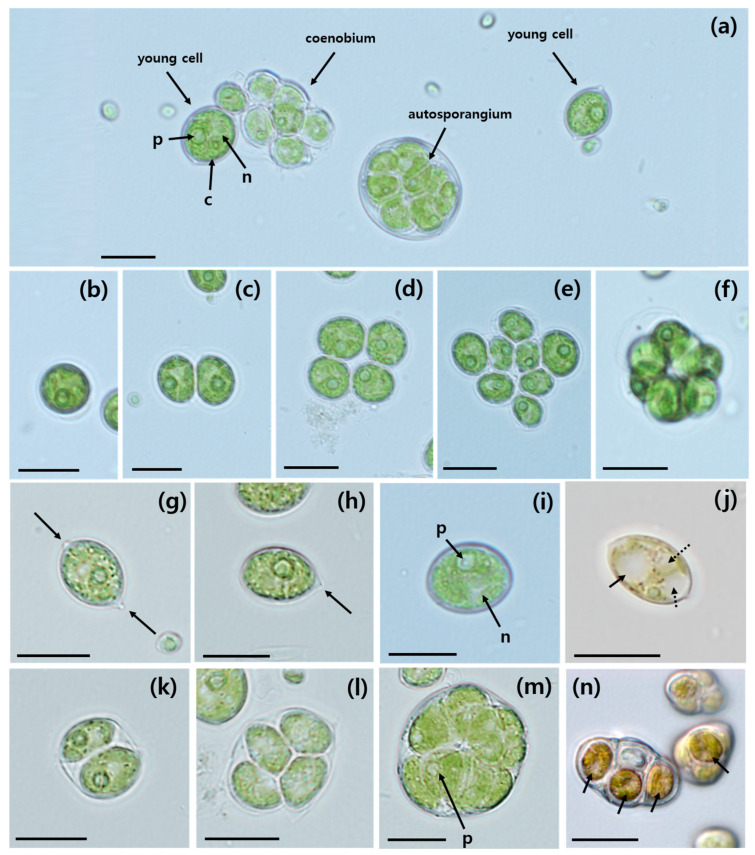
Light micrographs of *Asterarcys quadricellularis* AQYS21. (**a**) Young cells, autosporangium, and coenobium. A single pyrenoid (p), nucleus (n), and chloroplast (c) are shown. (**b**) A single-celled coenobium. (**c**) A two-celled coenobium. (**d**) A four-celled coenobium. (**e**) An eight-celled coenobium aligned in one plane. (**f**) An eight-celled coenobium aligned in two planes. (**g**) A young cell with pointed ends (arrows). (**h**) A young cell with a single pointed end. (**i**) A mature cell, with arrows indicating the nucleus (n) and pyrenoid (p). (**j**) A cell with one large vacuole (arrow) and several small vacuoles (dashed arrows). (**k**) An autosporangium with two autospores. (**l**) An autosporangium with four autospores. (**m**) An autosporangium with numerous autospores. The arrow indicates the pyrenoid (p). (**n**) Aging cells turned brick-red (arrows). Scale bars: (**a**–**n**) = 10 μm.

Young and mature cells, as well as autosporangia and coenobia, were observed under light microscopy (LM). These exhibited diversity in size and shape (Figure 1). In laboratory culture, coenobia consisting of 1, 2, 4, or 8 cells were arranged in one (Figure 1b–e) or two (Figure 1f) rows and were surrounded by a spherical mucilage envelope (Figure 1b–f, Table 1). Solitary young and mature cells were observed frequently. Young *A. quadricellularis* AQYS21 cells exhibited an asymmetric spindle-shaped morphology characterized by multiple pointed ends (Figure 1g, Table 1) or a single pointed end (Figure 1h, Table 1). The ranges (mean ± standard error, *n* = 20) of the cell lengths and widths were 9.2–15.8 µm (11.5 ± 0.3) and 6.6–11.8 µm (8.5 ± 0.3), respectively. In contrast, mature cells were sub-spherical in shape (Figure 1i). The ranges (mean ± standard error, *n* = 20) of the cell lengths and widths were 10.6–25.5 µm (19.8 ± 0.9) and 9.6–23.4 µm (18.1 ± 0.8), respectively.

Mature cells were slightly larger than young cells. Most young and mature cells had parietal chloroplasts with distinct pyrenoids (Figure 1a,i). Additionally, cells contain a single nucleus (Figure 1a,i). Some cells exhibited various sizes of vacuoles (Figure 1j). Asexual reproduction occurred through the formation of autospores; successive bipartition of the protoplast produced 2, 4, or more spores within the mother cell (Figure 1k–n, Table 1). A single pyrenoid was clearly visible in both young and mature cells as well as autospores (Figure 1a,i,m). Sexual reproduction was not observed in this species. In older cultures, *A. quadricellularis* AQYS21 accumulated yellow-red pigments, causing the algal mass to change from green to brick-red, as observed under LM (Figure 1n). This pigment accumulation resulted in aging cells typically exhibiting a brick-red color (Figure 1n).

**Figure 2:**
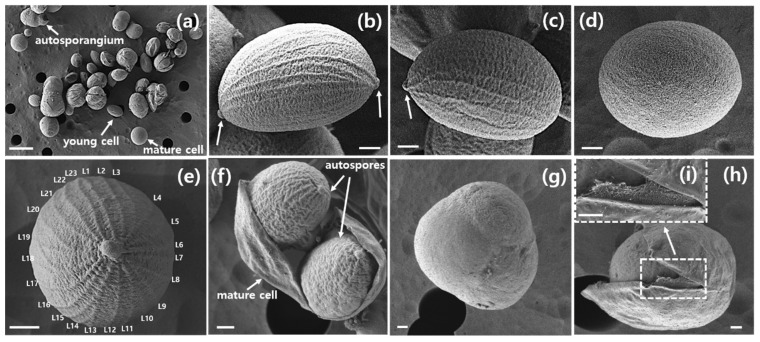
Scanning electron micrographs of the young, mature, and reproductive cells of *A. quadricellularis* AQYS21. (**a**) Various shapes and sizes of autosporangia, young cells, and mature cells at different stages of the life cycle. (**b**) A young cell with both ends pointed (arrows). (**c**) A young cell with one pointed end. (**d**) A mature cell. (**e**) A young cell with fine ribs on the surface. L1–L23 indicate the fine rib numbers. (**f**,**g**) Reproductive cells. (**h**) SEM of the ruptured mother cell wall. (**i**) Enlarged SEM of (**h**), showing the outer layer of the mother cell wall. Scale bars: (**a**) = 10 μm, (**b**–**i**) = 1 μm.

Scanning electron micrographs (SEM) of a group of young and mature cells, along with autosporangia of *A. quadricellularis* AQYS21 are shown in Figure 2. Figure 2a illustrates the diverse forms and sizes of young and mature cells, along with autosporangia of this strain. The SEM micrographs show the asymmetric, spindle-shaped morphology of young cells with pointed ends at both ends (Figure 2b) or at one end (Figure 2c). Mature cells appear sub-spherical in shape (Figure 2d, Table 1). As shown in the micrographs, the cells exhibited characteristic cell wall sculptures in the form of meridional fine ribs (Figure 2b–f). The meridional ribs (20–26) on the cell wall were barely detectable from apical views in LM but are clearly visible in SEM images (Figure 2e, Table 1). The ribs are prominent in young cells but gradually disappear with cell growth (Figure 2d). Furthermore, reproduction by 2–4 or more asexual autospores was frequently observed (Figure 2f,g). SEM micrographs showed that the mother cell wall was composed of multiple layers (Figure 2h,i).

**Figure 3:**
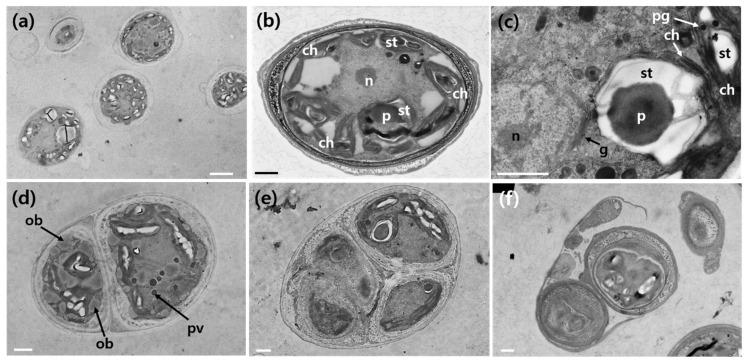
Transmission electron micrographs of *A. quadricellularis* AQYS21 cells in the exponential growth phase. (**a**) Various shapes and sizes at different stages of the life cycle. (**b**) A micrograph showing the chloroplast (ch), nucleus (n), and starch (st). (**c**) A micrograph showing a chloroplast (ch), Golgi apparatus (g), nucleus (n), pyrenoid (p), plastoglobule (pg), and starch (st). (**d**) TEM showing the formation of autospores, with two visible cells, along with oil bodies (ob) and vacuoles containing phosphate inclusions (pv). (**e**) TEM showing the formation of autospores, with four visible cells. (**f**) Release of autospores. Scale bars: (**a**) = 3 μm, (**b**–**f**) = 1 μm.

Transmission electron micrography (TEM) images illustrated various shapes, sizes, and the main ultrastructure of *A. quadricellularis* AQYS21 during the exponential growth phase (Figure 3). Thin sections prepared for TEM showed its primary cellular features, including the chloroplast (ch), Golgi apparatus (g), nucleus (n), oil body (ob), pyrenoid (p), plastoglobule (pg), vacuoles containing phosphate inclusions (pv), and starch (st) (Figure 3). Chloroplasts were observed mainly along the periphery of the cells (Figure 3b). Additionally, some chloroplasts surrounding the starch grains near the pyrenoid matrix were observed (Figure 3c). Two distinct types of starch deposition were identified, including solitary grains in the stroma and starch grains surrounding the pyrenoid matrix (Figure 3b,c, Table 1). However, the chloroplasts surrounding the starch grains near the pyrenoid matrix did not directly contact or penetrate the surface of the pyrenoid (Figure 3c). Oil bodies, plastoglobules, vacuoles containing phosphate inclusions, and reproductive cells were observed frequently within the cells (Figure 3c–f, Table 1).

### 2.2. Molecular Identification and Sequence Analysis

The combined length of the small-subunit (SSU) rDNA, the 28S rRNA gene region of the large-subunit (LSU) rDNA, *rbc*L (which encodes the large subunit of ribulose-1,5-bisphosphate carboxylase/oxygenase involved in photosynthesis), and *tuf*A (which encodes elongation factor Tu involved in protein synthesis) sequences in the newly isolated strain were 2885 nucleotides (GenBank accession numbers: OR910536, OR910537, OR916435, and OR916436; Table 2). Alignment results revealed that the SSU rDNA sequence of the *A. quadricellularis* AQYS21 isolate matched exactly with those from the *A. quadricellularis* strains Comas 77/75 (Escaleras de Jaruco, Cuba), KNUA020 (Daegu, Republic of Korea), FACHB-2316 (Zhengzhou, China), R-56 (Harbin, China), and TAU-MAC 3917 (Thessaloniki, Greece). However, strains A3 (Benha City, Egypt), BGLR5 (Muktsar, India), and an unidentified Indian strain exhibited variations, specifically, 1–5 base substitutions in the SSU compared to AQYS21 (Table 3). Moreover, *tuf*A in AQYS21 markedly differed from that in an unidentified Brazilian strain, with a two-base substitution in its sequence (Table 3).

**Figure 4:**
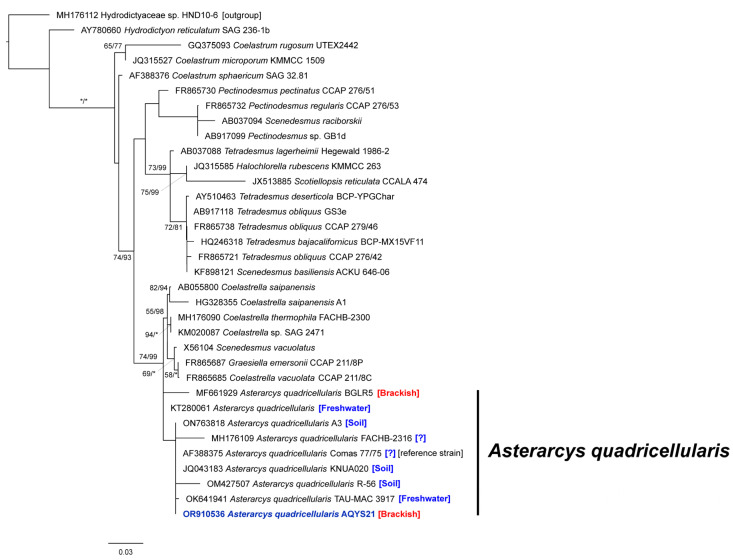
Maximum-likelihood and Bayesian inference phylogenetic tree based on 18S rDNA sequences. The values on each node indicate maximum-likelihood bootstrap percentages and Bayesian posterior probabilities (%). Bootstrap values <50 and Bayesian posterior probabilities <75 are not shown. The scale bar represents the number of nucleotide substitutions per site. * = 100.

In the phylogenetic tree based on SSU rDNA sequences, *A. quadricellularis* AQYS21 formed a large clade (i.e., *A. quadricellularis*) with strains BGLR5, A3, FACHB-2316, Comas 77/75, KNUA020, R-56, TAU-MAC 3917, and an unknown strain (GenBank accession no. KT280061) (Figure 4). Molecular characterization inferred from sequence analyses of SSU rDNA (Table 3, Figure 4) and *tuf*A (Table 3) confirmed that the isolate belongs to the *A. quadricellularis* group. Consequently, this microalga was identified as *A. quadricellularis* strain AQYS21, and it was deposited as a live culture at the National Marine Biodiversity Institute of Korea (MABIK) and the Korean Collection for Type Cultures (KCTC), where it is being actively preserved and maintained under accession numbers MABIK LP00000147 and KCTC 15413BP, respectively. Consequently, this microalga was identified as *A. quadricellularis* strain AQYS21, and it was deposited as a live culture at the National Marine Biodiversity Institute of Korea (MABIK) and the Korean Collection for Type Cultures (KCTC), where it is being actively preserved and maintained under accession numbers MABIK LP00000147 and KCTC 15413BP, respectively.

### 2.3. Verification of the Optimal Cultivation Conditions for the Isolated Strain

**Figure 5:**
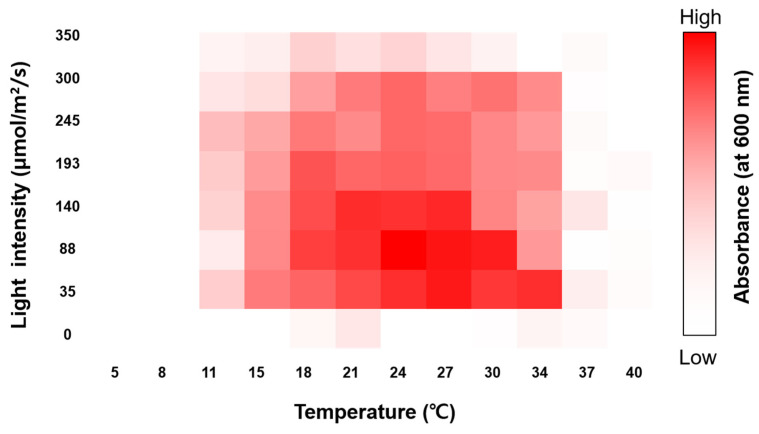
A heat map representing the screening of algal growth (optical density absorbance at 600 nm) response, as determined via PhotoBiobox analysis under different light intensities and temperatures.

Growth responses to different temperatures and light intensities were assessed under laboratory-scale conditions to verify the optimal cultivation conditions for the isolated algal strain. As shown in Figure 5, *A. quadricellularis* AQYS21 exhibited growth at 11–40 °C, with the highest growth rate observed at 24–30 °C and PFDs of 35–140 µmol m^−2^ s^−1^ (white LED). The specific optimal conditions were determined to be 24 °C and 88 µmol m^−2^ s^−1^. After optimization, algal growth was confirmed in a 60-mL T-flask (SPL, Republic of Korea) containing 25 mL of algal culture, achieving a biomass (dried cell weight) productivity of approximately 0.1132 g/L/d.

### 2.4. Proximate Composition and Fatty Acid Methyl Ester (FAME) Analysis, Along with the Evaluation of Biodiesel Properties

**Figure 6:**
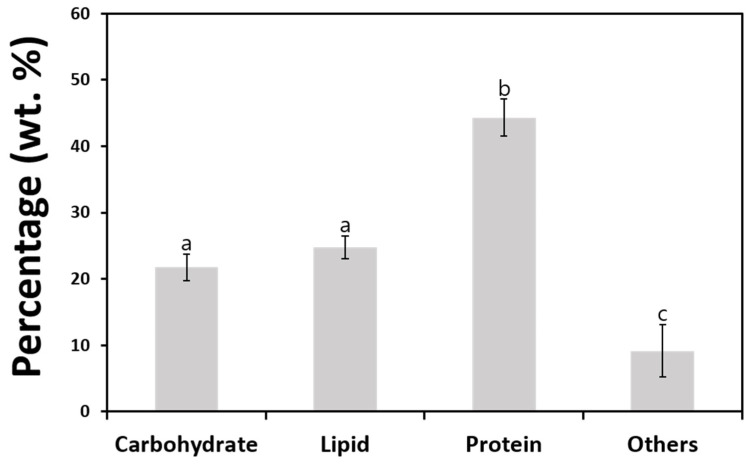
Total carbohydrate, lipid, and protein contents of *A. quadricellularis* AQYS21 cultivated in BG-11 medium under photoautotrophic conditions. Error bars indicate the mean ± S. D., and different letters represent significant differences (*p* < 0.05).

The analysis of dried algal biomass revealed different approximate compositions of total carbohydrates, lipids, and protein contents. As shown in Figure 6, total protein content was the highest among the three measured components, making up approximately 44.34% of the biomass. In contrast, lipid and carbohydrate contents accounted for 24.74% and 21.75% of the biomass, respectively.

Gas chromatography/mass spectrometry analysis revealed the fatty acid methyl ester (FAME) profiles of *A. quadricellularis* AQYS21 after 21 days of culture under vegetative growth conditions (exponential growth phase), and the results are presented in mass percentages (Table 4). The major FAMEs identified in this strain included palmitic acid (C_16:0_, 25.5%), oleic acid (C_18:1_ n-9, 13.3%), linoleic acid (C_18:2_ n-6, 5.98%), and α-linolenic acid (C_18:3_ n-3, 54.39%) (Table 4). Additionally, trace amounts of saturated fatty acids (SFAs) such as stearic acid (C_18:0_, 0.87%) were detected, accounting for 26.37% of the total fatty acid content (Table 4). In contrast, the total monounsaturated (MUFAs) and polyunsaturated (PUFAs) fatty acids accounted for 13.3% and 60.37%, respectively (Table 4).

*A. quadricellularis* AQYS21 exhibited a saponification value (SV) of 205.11, iodine value (IV) of 171.65, degree of unsaturation (DU) of 134.04, monounsaturated fatty acid (MUFA) content of 13.3%, polyunsaturated fatty acid (PUFA) content of 60.37%, long-chain saturation factor (LCSF) of 2.99, cold filter plugging point (CFPP) of −7.08, cetane number (CN) of 34.29, and oxidative stability (OS) value of 4.54 (Table 5).

## 3. Discussion

### 3.1. Morphological and Ultrastructural Characterization

The taxonomic, physiological, and biochemical characteristics of *A. quadricellularis* strain AQYS21 were analyzed to assess its potential as a commercially viable source of bioactive compounds and biofuels. The strain exhibited spherical to ellipsoidal cells, forming autospores with visible pyrenoid starch plates, consistent with the family Scenedesmaceae [46]. The coenobia of this isolate exhibited another characteristic of Scenedesmaceae; they were flat, slightly curved, and formed three-dimensional clusters [47]. These morphologies fit the criteria for classification into the family Scenedesmaceae, confirming the classification of *A. quadricellularis* AQYS21 within this family.

The morphological characteristics of *A. quadricellularis* AQYS21 closely aligned with the established description of the genus *Asterarcys*. The coenobia, consisting of 1, 2, 4, or 8 cells arranged in one or two rows and encased in a spherical mucilage envelope, reflect the typical cross or spherical alignment of this genus. The cells contain a single cup-shaped parietal chloroplast. Furthermore, asexual reproduction occurs through autospores, with 4–8 autospores per sporangium. These autospores are organized into coenobia and released through the rupture of the parental cell wall into two pieces that remain visible for some time. The frequent observation of solitary cells in both young and mature stages is consistent with the report by Comas Gonzalez [31].

The cell dimensions of *A. quadricellularis* AQYS21 exhibit a range consistent with the genus *Asterarcys*. The length and width of young cells fell within the described size range for *Asterarcys* cells, which is typically 7–18 × 7–18 µm [31]. Furthermore, the observed diversity in cell shapes, including broadly oval, irregularly spherical, trapezoidal, and triangular forms, aligns with the morphology of the genus *Asterarcys*.

*A. quadricellularis* AQYS21 exhibits shared morphological, ultrastructural, and reproductive traits with *A. quadricellularis* strains from Guatemala (Guatemala strain), Greece (TAU-MAC 3917), and India (PUMCC 5.1.1) (Table 1). Therefore, our isolate was conclusively identified as *A. quadricellularis* based on these shared traits.

There have been no reports of *A. quadricellularis* strains showing ribs on their cell surfaces to date (Table 1). However, young cells of the *A. quadricellularis* AQYS21 strain displayed approximately 20–26 distinct meridional ribs in the present study (Figure 2e).

Several similar genera of green coccoid microalgae also exhibit ribs on their cell surfaces. For instance, *Coelastrella* has 16–40 ribs [48], *Graesiella* has a fine network of ribbing [49], *Scotiellopsis* has meridional ribs from pole to pole [50], and *Enallax* has longitudinal ribs from pole to pole [51,52]. However, *A. quadricellularis* AQYS21 is distinct from these genera, owing to its characteristic 2-, 4-, and 8-celled coenobia embedded in mucilage. Furthermore, young *A. quadricellularis* AQYS21 cells demonstrate an asymmetric spindle-shaped form, with either pointed ends or a single pointed end—a morphology not previously noted in other *A. quadricellularis* strains (Table 1). Therefore, future studies should investigate whether these morphological features, including the pointed ends and ribs on the cell surfaces, are present in other *A. quadricellularis* strains.

Starch deposition in *A. quadricellularis* AQYS21 was observed in two distinct forms, with solitary grains in the stroma and starch grains surrounding the pyrenoid matrix. Notably, the chloroplasts surrounding the starch grains near the pyrenoid matrix did not directly contact or penetrate the surface of the pyrenoid. The pyrenoids and their associated starch components are crucial diagnostic features for identifying coccal green algae [53]. In previous studies, the ultrastructure of the pyrenoid, including the number and position of starch grains, has been used to classify various species of green algae within the genera *Chlorococcum* and *Tetracystis* [54,55]. Furthermore, the pyrenoid matrix and the thylakoids traversing the matrix have been shown to exhibit species-specific characteristics [56,57]. The observations in *A. quadricellularis* AQYS21 suggest that the starch deposition patterns, particularly the presence of solitary grains in the stroma and starch grains surrounding the pyrenoid matrix, could serve as important morphological traits for future taxonomic studies. The lack of direct contact between the thylakoids and the pyrenoid surface is another notable feature that could aid species differentiation.

The color change observed during the senescence of *A. quadricellularis* AQYS21 cultures (Figure 1n), where cells transitioned from green to brick-red as they aged, is likely due to the accumulation of secondary carotenoids, as is common in other carotenoid-producing microalgae. These pigments, such as β-carotene and astaxanthin, are typically synthesized in response to environmental stressors such as nutrient depletion, high light intensity, or oxidative stress. Secondary carotenoids function as antioxidants and light filters, protecting the photosynthetic apparatus from photodamage by reducing excess light absorption. While these carotenoids do not directly participate in photosynthesis, they serve as an adaptive mechanism, often resulting in the red or orange coloration of cells, as observed in species like *Haematococcus* and *Dunaliella* [58]. In *A. quadricellularis* AQYS21, the color change suggests similar carotenoid accumulation. However, due to the absence of pigment composition analysis in the present study, we cannot conclusively determine the specific carotenoids responsible. Future research should focus on pigment analysis to identify the types of secondary carotenoids produced under various environmental conditions. Such research will also provide deeper insights into the taxonomic and physiological significance of these pigments.

### 3.2. Molecular-Phylogenetic Characterization

In this study, we performed taxonomic classification using molecular marker sequencing to support the morphological identification. The SSU rDNA sequence of *A. quadricellularis* AQYS21 was identical to those of several other strains, including the reference strain *A. quadricellularis* Comas 77/75 and strains KNUA020, FACHB-2316, R-56, and TAU-MAC 3917 (Table 3). Phylogenetic analyses based on the SSU rDNA region confirmed that strain AQYS21 belongs to *A. quadricellularis* (Figure 4). Furthermore, the *tuf*A chloroplast gene of *A. quadricellularis* AQYS21 showed a high similarity (99.8%) to that in an *A. quadricellularis* strain from Brazil (Accession no. KT429436), as determined through direct sequence comparison (Table 3). This green alga has consequently been identified as *A. quadricellularis* based on the genetic results.

### 3.3. Ecological and Growth Characteristics, Including Adaptability

Before this study, *A. quadricellularis* strains were reported to inhabit freshwater environments in Egypt, Greece, India, Qatar, Brazil, and South Africa [36,40,59,60,61,62]. Additionally, some strains were found in soil in Korea (strain KNUA020), China (strain R-56), and Egypt (strain A3) [33,34]. Thus, *A. quadricellularis* strains primarily inhabit freshwater and soil environments (Table 3, Figure 4). The presence of *A. quadricellularis* strains in brackish waters, such as the BGLR5 strain in India [63], is rarely detected. In this study, the AQYS21 strain was isolated from brackish water (15.3 PSU) in Korea. Our findings suggest that *A. quadricellularis* is distributed across various habitats, including soil, freshwater, and brackish water.

Optimal physical conditions, such as temperature and light intensity, are crucial for maximizing the growth of microalgae [64,65,66]. In this study, *A. quadricellularis* AQYS21 grew well at 11–40 °C, with the highest growth observed between 24 °C and 30 °C and at a PFD range of 35–140 µmol m^−2^ s^−1^ (Figure 5). The optimal growth temperature range for *A. quadricellularis* AQYS21 is slightly lower than the reported optimal range of 30–35 °C for the PUMCC 5.1.1 strain [35]. However, *A. quadricellularis* AQYS21 exhibited robust growth even at higher temperatures in the present study, suggesting that it can adapt to varying environmental conditions.

Light intensity substantially affects photosynthesis and biomass production in microalgae [65]. *A. quadricellularis* AQYS21 showed adaptability across a wide range of light intensities (Figure 5). When combined with its broad temperature adaptability, this flexibility suggests that *A. quadricellularis* AQYS21 can thrive under various environmental conditions with differing light availability. Although light intensity is a key factor in photosynthesis, the adaptability of this strain to both light and temperature may contribute to its ability to inhabit diverse ecosystems, potentially explaining its global distribution.

The adaptability of this strain suggests that it could be highly effective in various biotechnological applications, making it particularly promising for numerous industrial applications. Thus, future research aimed at optimizing the cultivation conditions and genetic traits of this strain could further enhance its industrial viability.

### 3.4. Carbohydrate and Lipid Contents

The total carbohydrate and lipid contents are crucial indicators of biofuel productivity, as algal carbohydrates and lipids can be converted into bioethanol and biodiesel through fermentation and transesterification, respectively. The total carbohydrate and lipid contents of *A. quadricellularis* AQYS21 demonstrated notable potential at approximately 21.75% and 24.74%, respectively.

Biodiesel production from microalgal lipids is facilitated by transesterification, wherein algal lipids are mixed with alcohol in the presence of a catalyst. Therefore, lipid productivity is a key factor in assessing the feasibility of microalgae for biodiesel applications [67]. Various green microalgal species, such as *Botryococcus braunii*, *Tetradesmus obliquus*, and *Grasiella emersonii*, have demonstrated high lipid yields, with some achieving up to 86% of dried cell weight under optimized conditions. However, these species typically exhibit lipid accumulation of approximately 13–31% under normal conditions [68]. A higher total lipid content (24.74%) was observed under normal conditions in the present study, suggesting a potential for further optimization. Therefore, future studies should focus on optimizing growth conditions, such as nutrient deficiency or heterotrophic cultivation, to enhance lipid accumulation and improve the biomass productivity of *A. quadricellularis* AQYS21 for biodiesel production. These strategies have significantly increased lipid content in microalgae, thus maximizing their utility as a biofuel feedstock in previous studies [68].

### 3.5. Fatty Acid Composition and Biodiesel Properties

High levels of palmitic acid, oleic acid, linoleic acid, and α-linolenic acid were detected in *A. quadricellularis* AQYS21 (Table 4). Of these, the percentage of palmitic acid was particularly high. Palmitic acid is an important saturated fatty acid used in food additives, soap-making, waterproofing materials, and as a base in organic synthesis [69]. Due to its industrial significance, the global market value of palmitic acid was approximately USD 253.7 million in 2023 and is expected to reach USD 314.9 million by 2032. Driven by a compound annual growth rate (CAGR) of 2.4% from 2024 to 2032, this growth is attributed to increased usage of food products, ongoing research and development, and expanded industrial applications [70].

Palmitic acid is highly valued in biodiesel production, owing to its high CN, which indicates superior combustion quality [71]. The presence of considerable amounts of palmitic acid and other SFAs, such as stearic acid (C_18:0_), in microalgal lipids contributes to a higher CN and stability, making these lipids ideal for biodiesel production [72,73]. The palmitic acid content in AQYS21 was higher than that in other *A. quadricellularis* strains, such as KNU020 (15.3%) and those from Benha and Menoufia, Egypt (17.17% and 20.54%). However, it is lower than that in the strain from Harbin, China (56.24%) (Table S1). The palmitic acid content of AQYS21 surpasses that of other oil sources, including jatropha (13.4%), karanja (7.4%), mahua (21.5%), and rapeseed (3.49%), but is lower than that of palm oil (47.9%) (Table S1). Considering the higher photosynthetic efficiency and oil production rates of microalgae than those of terrestrial plants [13], *A. quadricellularis* AQYS21 emerges as a promising alternative raw material for biodiesel production. The combination of high palmitic acid content with the advantages of microalgal cultivation positions AQYS21 as a sustainable and efficient source of biofuels and various industrial products.

Essential PUFAs, such as α-linolenic acid (ALA), play a crucial role in human health. They affect the regulation of inflammation, cardiovascular health, and cancer prevention [74]. As mammals, including humans, cannot synthesize essential PUFAs such as ALA, they must be obtained through the diet from sources such as fish, plants, and microalgae [75,76,77]. The microalgae *A. quadricellularis* AQYS21 has exceptionally high ALA content, which constitutes 54.39% of its total fatty acids (Table S1). This ALA content is substantially higher than those reported in other *A. quadricellularis* strains, including KNU020 (41.2%) and those from Benha, Egypt (24.32%); Menoufia, Egypt; and China, where no ALA was detected. Moreover, the ALA levels in AQYS21 exceed those found in other microalgae species, such as *Dunaliella salina* LIMS-PS-1511 (31.7%), *Graesiella emersonii* GEGS21 (22.1%), *Chlamydomonas hedleyi* MM0020 (16.4%), and *Microglena monadina* NFW3 (53.01%), further highlighting its superiority (Table S1). Furthermore, the ALA content of AQYS2 is significantly greater than that of second-generation oil sources such as jatropha (0.2%), palm, rapeseed, mahua, and karanja, which show either negligible or no detectable ALA content (Table S1). The high concentration of ALA in AQYS21 emphasizes its potential as a superior alternative to traditional plant-based sources of essential PUFAs. The ability of this strain to produce large amounts of ALA, coupled with the environmental benefits of microalgal cultivation, positions *A. quadricellularis* AQYS21 as a promising resource for nutraceutical and pharmaceutical applications, offering a sustainable and effective source of essential fatty acids for global markets.

To evaluate the biodiesel quality of *A. quadricellularis* AQYS21 compared to terrestrial plants and other microalgae, we analyzed their biodiesel properties based on FAME profiles (Table 5). Both lipid content and fatty acid composition are crucial when determining the suitability of algal biomass for biofuel production, as these factors markedly impact the FAME profile, which in turn determines biodiesel quality [76,78]. Key biodiesel properties such as CN, IV, and OS are critical for assessing diesel engine performance, consequently affecting combustion quality, storage stability, and cold-flow characteristics [79,80,81]. Comparison of these properties between *A. quadricellularis* AQYS21 and other species provides valuable insights into its potential for biodiesel production.

The IV of *A. quadricellularis* AQYS21 (171.65) exceeded the maximum limit of 120 set by the EN14214 standard for biodiesel, indicating a higher degree of unsaturation that may affect oxidative stability and cold-flow properties (Table 5). In contrast, terrestrial plant oils such as palm and rapeseed oil have lower IVs of 48.05 and 115.07, respectively, suggesting a more balanced fatty acid profile suitable for biodiesel. However, the IV of AQYS21 is within the range of other microalgae, such as *Mychonastes homosphaera* UTEX 2341 (162.79), suggesting that *A. quadricellularis* AQYS21 could still be considered for specific applications where higher IVs are acceptable (Table 5).

The CN of AQYS21 (34.29) was below the minimum requirements of 51 and 47 set by the EN14214 and ASTM D6751-02 standards, respectively (Table 5). Although AQYS21 may not meet the ignition quality standards for biodiesel in Europe and the US, it could be considered for blending with other biodiesel sources with higher CN values to achieve an acceptable fuel mix [82]. Terrestrial oils like mahua and palm showed higher CNs of 59.55 and 63.5, respectively, indicating better ignition properties.

The AQYS21 OS value (4.54) was below the EN14214 requirement of 6 but above the ASTM D6751-02 requirement of 3 (Table 5). This suggests that while AQYS21 has moderate resistance to oxidation, making it suitable for certain biodiesel applications, it may require antioxidants or blending with more stable oils to enhance its storage properties. *Mychonastes homosphaera* UTEX 2341 exhibited a markedly higher OS value (23.28) than AQYS21, indicating a significant advantage in storage stability.

In conclusion, although *A. quadricellularis* AQYS21 exhibits certain limitations in its biodiesel properties, such as a high IV and lower CN, its moderate OS and potential for blending make it a promising candidate for biodiesel production. Further optimization through cultivation techniques and biotechnological interventions could enhance its fuel properties, making it more competitive with other biodiesel sources.

This study elucidated the fatty acid composition of *A. quadricellularis* AQYS21, revealing high percentages of palmitic acid, oleic acid, linoleic acid, and ALA. These findings highlight the potential of this strain for various industrial applications, particularly in biofuel production. Our robust morphological and molecular data provide a solid foundation for verifying the identity and purity of this strain, paving the way for further applied research.

By registering *A. quadricellularis* AQYS21 with the National Marine Biodiversity Institute of Marine Bio-Bank, we have established a critical resource for future research and industrial applications. Further studies aimed at optimizing the cultivation conditions and genetic traits of this microalga could enhance its application potential in the bioindustry, leading to the sustainable production of commercially valuable biochemicals and biofuels.

## 4. Materials and Methods

### 4.1. Sample Collection and Isolation

**Figure 7:**
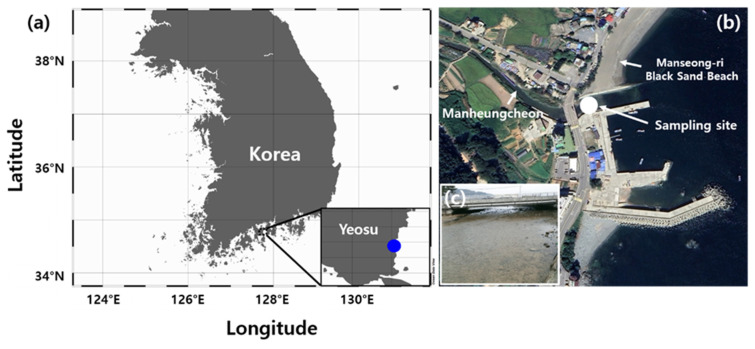
Location and photographs of the sampling site in the Manseong-ri Black Sand Beach, Republic of Korea. (**a**) A map of the sampling site and location of the site in the Manseong-ri Black Sand Beach. Located in the northeastern coastal area of Yeosu City in Jeollanam-do, this bathing beach stretches 735 m in length and 50 m in width. Part of the beach forms a buffer zone where freshwater from the Manheungcheon Stream merges with seawater. (**b**) The environment surrounding the Manseong-ri Black Sand Beach in the Republic of Korea; image acquired using Google Earth. (**c**) The sampling site of *Asterarcys quadricellularis* AQYS21.

Plankton samples were collected from brackish water near Manseong-ri Black Sand Beach, Yeosu-si, Jeollanam-do, Republic of Korea (34°46′28″ N, 127°44′41.0″ E) in May 2021. The water temperature was 23.2 °C and the practical salinity unit (PSU) was 15.3 (Table 2 and Figure 7). The sampling site at Manseong-ri Black Sand Beach experiences substantial fluctuations in salinity, owing to continuous freshwater inflow from the Manheungcheon Stream and the influence of tidal currents. The water sample was mixed with an equal volume of BG-11 medium (Sigma-Aldrich, St. Louis, MO, USA) and left to stand for two days to simulate eutrophication. BG-11 medium was selected due to its well-established effectiveness in supporting the growth and lipid production of various microalgae, including both freshwater and marine species, as noted in recent studies [83]. Cultures were aseptically isolated using a streak plate technique on BG-11 medium containing 1.5% agar. The plates were incubated in a culture room at 28 °C under cool fluorescent light (~60 μmol m^−2^ s^−1^) with a 14 h/10 h light/dark cycle until green microalgal colonies formed. Single colonies were aseptically transferred to fresh BG-11 plates; this step was repeated until a pure culture was obtained [84]. The axenic (bacteria-free) status of the algal culture was confirmed by spreading 50 μL of liquid culture of the single colony onto 1.5% Luria–Bertani (LB) agar plates (Sigma-Aldrich), followed by verification using 16S rRNA-based colony PCR to detect any bacterial contamination [85]. The isolated algal strain was maintained at 28 °C in a plant growth chamber (JSR, Gongju, Republic of Korea), with shaking at 150 rpm under continuous illumination of 60 μmol photons m^−2^ s^−1^ from cool-white, fluorescent lights.

### 4.2. Morphological Identification

The morphology of living cells grown photosynthetically was examined using an inverted microscope (CKX53, Olympus, Tokyo, Japan). The length and width of the live cells were measured with a digital camera (Zeiss AxioCam MRc5; Carl Zeiss, Göttingen, Germany).

For field emission scanning electron microscopy (FE-SEM), 10-mL aliquots of cultures, at a density of approximately 2 × 10^6^ cells mL^−1^, were fixed in a commercially available 4% (*w*/*v*) osmium tetroxide (OsO_4_; Electron Microscopy Sciences, Hatfield, PA, USA) solution, which was diluted to a final concentration of 1% (*w*/*v*) by mixing with the culture medium. The fixation was carried out for 10 min without using any buffer system. The fixed cells were collected on polycarbonate membrane filters with 3 µm pores (Whatman Nuclepore Track-Etched Membranes; Whatman, Kent, UK) and washed thrice with distilled water. The membranes with the attached cells were dehydrated in a graded ethanol series (10, 30, 50, 70, 90, and 100% ethanol), followed by two changes in 100% ethanol (Merck, Darmstadt, Germany). Next, they were immediately dried using an automated critical point dryer (EM CPD300, Leica, Wetzlar, Germany) with CO_2_ for critical point drying. The dried filters were mounted on an aluminum stub (Electron Microscopy Sciences) using copper conductive double-sided tape (Ted Pella, Redding, CA, USA) and coated with gold using an ion sputter (MC1000, Hitachi, Tokyo, Japan). The cells and surface morphologies were observed using a high-resolution Zeiss Sigma 500 VP FE-SEM (Carl Zeiss).

Cells in the exponential growth phase were transferred to a 10 mL tube and fixed in 2.5% (*v*/*v*) glutaraldehyde (final concentration) for 1.5 h for TEM. The tube contents were placed in a 10 mL centrifuge tube and concentrated at 1610× *g* for 10 min in a centrifuge (VS-5500; Vision, Bucheon, Republic of Korea). The resulting pellet was transferred to a 1.5 mL tube and rinsed several times with 0.2 M sodium cacodylate buffer at pH 7.4 (Electron Microscopy Sciences). The cells were then post-fixed with 1% (*w/v*) OsO_4_ prepared in deionized water for 90 min. The pellet was embedded in agar before dehydration in a graded ethanol series (50, 60, 70, 80, 90, and 100% ethanol), followed by two changes in 100% ethanol. The material was then embedded in Spurr’s resin (Electron Microscopy Sciences). Sections were prepared using an EM UC7 ultramicrotome (Leica) and stained with 3% (*w/v*) aqueous uranyl acetate (Electron Microscopy Sciences), followed by 0.5% (*w/v*) lead citrate (Electron Microscopy Sciences) [86]. The sections were visualized using TEM (Sigma 500/VP TEM; Carl Zeiss).

### 4.3. Molecular Identification

Genomic DNA (gDNA) was extracted using an AccuPrep Genomic DNA Extraction Kit (Bioneer, Daejeon, Republic of Korea) according to the manufacturer’s instructions for molecular analysis. The primers used to amplify each marker gene are listed in Table 6. The reaction mixtures for PCR amplification comprised 5 µL of 10× F-Star Taq reaction buffer, 1 µL of 10 mM dNTP mix, 0.02 µM of primers, 0.25 µL of 5 U µL^−1^ BioFACT F-Star Taq DNA polymerase (BioFACT Co., Ltd., Daejeon, Republic of Korea), 38.75 µL of UltraPure DNAse/RNAse-Free Distilled Water (Invitrogen, Carlsbad, CA, USA), and 3 µL of the DNA template (ca. 10–30 ng DNA). PCR amplification was performed on an Eppendorf Mastercycler PCR machine (Eppendorf, Hamburg, Germany) under the following thermal cycling conditions: pre-denaturation at 94 °C for 5 min, followed by 35 cycles of 94 °C for 1 min, the selected annealing temperature (AT) for 1 min, 72 °C for 1 min, and final extension at 72 °C for 5 min. The AT of the primers was determined through gradient PCR. We optimized the ATs as follows: 52.4 °C (EukA-G18R), 52.4 °C (570F-EukB), 53.0 °C (D1R-LSUB), 52.4 °C (*rbc*L-192-*rbc*L-657), and 50.0 °C (Tuf-F-Tuf-R). PCR products were purified using the AccuPrep PCR Purification Kit (Bioneer) and subjected to Sanger sequencing (Macrogen, Daejeon, Republic of Korea). Nucleotide sequences were identified using the National Center for Biotechnology Information (NCBI) Basic Local Alignment Search Tool.

Alignments, as well as phylogenetic and molecular evolutionary analyses of the obtained sequences, were conducted utilizing Geneious Prime v.2022.2.2 (Biomatters Ltd., Auckland, New Zealand). This analysis incorporated various assemblages, drawing on data from other species available in the NCBI GenBank database. Bayesian analyses were run using MrBayes v.3.2.7 [93,94] with the default GTR + G + I model to determine the best available model for the data of each region. Four independent Markov chain Monte Carlo runs were performed as described by Kang et al. [95] for all sequence regions. Moreover, maximum-likelihood analyses were conducted using RAxML v.8.2.10 [96]. Two hundred independent free inferences were allowed, and the –# option was used to identify the best tree. Bootstrap values were calculated with 1000 replicates using the same substitution model.

### 4.4. Determination of Optimal Culture Conditions

Routine serial sub-culturing was performed on BG-11 agar slants to maintain a pure culture of *A. quadricellularis* AQYS21. A single colony of this strain was streaked onto BG-11 agar plates and incubated for 21 days. Next, a single colony was cultured in BG-11 medium, and an optimal culture test was conducted at a laboratory scale. Optimal temperature and illumination analyses were conducted simultaneously using a PhotoBiobox [97]. A 200 µL algal culture aliquot was injected into 96-well black/clear bottle plates and covered with well plate sealing film. After incubation for 72 h in PhotoBiobox controlled at 5–40 °C and 0–350 µmol m^−2^ s^−1^, optical density was measured at 600 nm using a Synergy II microplate reader (Biotek, Winooski, VT, USA). The specific growth rate (μ) was calculated using the formula μ = (ln A_2_ − ln A_1_)/(t_2_ − t_1_), where A_1_ and A_2_ represent the optical density values at t = 0 and t = 72 h, respectively. The calculated growth rates were then visualized as a heat map using Microsoft Excel (Microsoft Corp., Redmond, WA, USA).

### 4.5. Determination of Total Lipid, Carbohydrate, and Protein Contents

Total carbohydrate content was evaluated by resuspending 5–6 mg of lyophilized algal cells in 25 mL of distilled water. A 1-mL aliquot of this suspension was mixed with 1 mL of 5% phenol solution, followed by the addition of 5 mL of sulfuric acid as described by DuBois et al. [98]. The samples were cooled in a cold water bath, and optical density was measured at 488 nm using a Synergy II microplate reader (BioTek, Winooski, VT, USA). Carbohydrate content was then calculated using a glucose standard curve.

Total protein content was determined by converting the measured total nitrogen content (wt. %). Nitrogen content was quantified through elemental analysis at 950 °C using a Leco N determinator (model FP-528; LECO Corporation, St. Joseph, MI, USA), with ultra-high-purity oxygen and helium as the combustion and carrier gases, respectively. The nitrogen-to-protein conversion factors used to estimate crude protein content followed the guidelines of Mariotti et al. [99]. The total lipid content was extracted from dried algal biomass using a methanol-chloroform solvent mixture, as described by Kim et al. [100].

### 4.6. Analyses for Fatty Acid Composition of Lipids and Their Biodiesel Properties

Lipid extraction was performed using a modified version of the Bligh–Dyer method, as described by Breuer et al. [101], after 21 days of culture in BG-11 medium under photoautotrophic conditions and vegetative growth (exponential growth phase). The FAME composition was analyzed using a 7890A gas chromatograph equipped with a 5975C mass selective detector (Agilent Technologies, Santa Clara, CA, USA) as described in our previous study [60]. Compound identification was performed by matching the mass spectra with those in the Wiley/NBS registry of mass spectral data; a match value of >90% was considered valid. Biodiesel properties, including SV, IV, DU, MUFA, PUFA, LCSF, CFPP, CN, and OS, were calculated based on the FAME profiles, as described by Islam et al. [79].

### 4.7. Statistical Analysis

All experiments were conducted in triplicate; data are expressed as the mean ± standard deviation. Data analysis was carried out using a one-way analysis of variance, followed by Tukey’s HSD test, to assess differences between means utilizing SPSS v.14.0 software (IBM, SPSS Inc., Armonk, NY, USA). Prior to conducting the analysis of variance and Tukey’s HSD test, the normality of the data was assessed using the Shapiro-Wilk test. *p* < 0.05 was considered statistically significant, and differences between values are indicated by distinct letters.

## Figures and Tables

**Table 1:** Comparison of morphological and ultrastructural characteristics among *A. quadricellularis* strains.

Character Traits	AQYS21	Guatemala	TAU-MAC 3917	PUMCC 5.1.1
Strain locality	Republic of Korea	Guatemala	Greece	India
Coenobia	Present, 1-, 2-, 4-, 8-celled coenobia embedded in mucilage	Present, 1-, 2-, 4-, 8-celled coenobia embedded in mucilage	Present, cells surrounded by a mucilage envelope	Present, 2–4 or more cells within a spherical mucilage envelope
Shape of young/mature cells	Asymmetric spindle-shaped	ND	ND	ND
**Pointed position of young cells**	**Both ends or one end pointed**	ND	ND	ND
Cell shape of mature cells	Sub-spherical to spherical shaped	ND	Spherical shaped *	Spherical shaped *
Pyrenoid	Present, surrounded by the starch grains	Present	Present, surrounded by the starch grains *	Present, surrounded by the starch grains *
Asexual reproduction	Present, mainly 2–8 autospores. Some cells have more than eight	4–8	Present, some cells have more than eight	ND
**Number of cell wall ribs**	**20–26**	ND	ND	ND
Cytoplasmic oil bodies	Present	ND	Present	ND
Reference	This study	[43]	[40]	[35]

ND, information not available; * Not mentioned but observed from figures.

**Table 2:** Strain, location of collection (LC), water temperature (T, °C), salinity (S, PSU), and GenBank accession numbers for marker genes of *Asterarcys quadricellularis* AQYS21 isolated from Manseong-ri Black Sand Beach, Yeosu-si, Jeollanam-do, Republic of Korea.

Species	Strain	LC	Date	T (℃)	S (PSU)	Marker Gene	Amplicon Length (bp)	GenBank Accession Number
*A. quadricellularis*	AQYS21	Manseong-ri Black Sand Beach	May 2021	23.2	15.3	SSU	923	OR910536
						LSU	567	OR910537
						rbcL	428	OR916435
						tufA	967	OR916436

**Table 3:** Comparison of small-subunit rDNA and *tuf*A sequences of *A. quadricellularis* AQYS21 isolated from the Manseong-ri Black Sand Beach of Korea with those of other strains.

Collection Location	Strain Habitat (Isolation Source)	Strain Name	GenBank Accession Number	*Asterarcys quadricellularis* AQYS21 *
Escaleras de Jaruco, Cuba	ND	Comas 77/75	AF388375	0 (0)
Daegu, Republic of Korea	Soil	KNUA020	JQ043183	0 (0)
Zhengzhou, China	ND	FACHB-2316	MH176109	0 (0)
Harbin, China	Soil	R-56	OM427507	0 (0)
Thessaloniki, Greece	Freshwater	TAU-MAC 3917	OK641941	0 (0)
Benha City, Egypt	Soil	A3	ON763818	1 (0.1)
Chennai, India	Freshwater	ND	KT280061	1 (0.1)
Muktsar, India	Brackish	BGLR5	MF661929	5 (0.5)
Brazil	Freshwater	ND	KT429436 **	2 (0.2)

* The numbers indicate the number of base pairs that differ from *A. quadricellularis* AQYS21 between strains. The numbers in parentheses indicate dissimilarity (%), including gaps. ND, information not available. ** The GenBank accession number is the sequence of *tuf*A gene.

**Table 4:** The lipid profile of *A. quadricellularis* AQYS21.

Component	Content (%)	Note
Palmitic acid (C_16:0_)	25.5	SFA (major)
Stearic acid (C_18:0_)	0.87	-
Oleic acid (C_18:1_ n-9)	13.3	ω-9 MUFA (major)
Linoleic acid (C_18:2_ n-6)	5.98	ω-6 PUFA (major)
α-linolenic acid (C_18:3_ n-3)	54.39	ω-3 PUFA (major)
Total saturated fatty acids	26.37	
Total monounsaturated fatty acids	13.3	
Total polyunsaturated fatty acids	60.37	

SFA, saturated fatty acids; MUFA, monounsaturated fatty acids; PUFA, polyunsaturated fatty acids.

**Table 5:** Biodiesel properties calculated from the FAME compositions of the isolated algal strain and other crops, and biodiesel standard EN 14214 [44] and ASTMD6751-02 [45].

Source	SV (mg KOH/g)	IV (g I_2_/100 g)	DU	MUFA (%)	PUFA (%)	LCSF	CFPP (°C)	CN	OS (h)
Jatropha	190.98	105.42	122.1	37.3	42.4	4.54	−2.21	51.16	5.37
Karanja	184.05	94.22	105.2	65.6	19.8	2.64	−8.18	54.76	8.55
Mahua	191.58	67.72	78.62	39.1	19.76	11.65	20.12	59.55	8.56
Palm	194.82	48.05	55.7	37.04	9.33	6.91	5.22	63.5	15.23
Rapeseed	188.61	115.07	125.46	64.4	30.53	0.77	−14.05	49.35	6.45
*A. quadricellularis* AQYS21	205.11	171.65	134.04	13.3	60.37	2.99	−7.08	34.29	4.54
*Chlamydomonas hedleyi* MM0020	95.6	62.09	55.0	2.6	26.2	2.43	−8.84	89.42	7.09
*Chlorella salina* MM0063	132.19	100.26	85.0	2.6	41.2	2.75	−7.84	65.03	5.45
*Coelastrum microporum* IBL-C119	181.83	82.61	84.64	45.24	19.7	4.02	−3.84	57.73	8.58
*Dunaliella salina* LIMS-PS-1511	121.29	95.41	78.3	3.7	37.3	2.73	−7.9	69.83	5.75
*Graesiella emersonii* GEGS21	204.86	131.06	121.3	22.5	49.4	3.05	−6.89	43.45	4.98
*Haematococcus lacustris*	162.7	98.86	95.81	20.13	37.84	3.82	−4.46	57.6	5.75
*Microglena monadina* NFW3	188.54	166.15	138.24	3.28	67.48	2.71	−7.97	37.86	4.34
*Mychonastes homosphaera* UTEX 2341	142.74	162.79	98.9	23.9	37.5	1.45	−11.92	47.91	23.28
*Jaagichlorella luteoviridis* MM0014	157.6	109.69	110.7	7.1	51.8	2.77	−7.77	56.25	4.87
*Tetradesmus obliquus* MM0026	138.92	98.63	85.4	18.4	33.5	2.45	−8.78	63.4	6.11
EN14214	-	≤120	-	-	-	-	≤−20~5	≥51	≥6
ASTM D6751-02	-	-	-	-	-	-	-	≥47	≥3

SV, saponification value; IV, iodine value; DU, degree of unsaturation; MUFA, monounsaturated fatty acid; PUFA, polyunsaturated fatty acid; LCSF, long-chain saturation factor; CFPP, cold filter plugging point; CN, cetane number; OS, oxidative stability.

**Table 6:** Primers used to amplify the small-subunit (SSU) rDNA, the 28S rRNA gene region of the large-subunit (LSU) rDNA, and the *rbc*L and *tuf*A genes of *Asterarcys quadricellularis* AQYS21.

Primer Name	Primer Region	Sequence (5′-3′)	Reference
EukA	Forward, SSU	AACCTGGTTGATCCTGCCAG	[87]
G18R	Reverse, SSU	GCATCACAGACCTGTTATTG	[88]
570F	Forward, SSU	GTAATTCCAGCTCCAATAGC	[89]
EukB	Reverse, SSU	TGATCCTTCTGCAGGTTCACCTAC	[87]
D1R	Forward, LSU	ACCCGCTGAATTTAAGCATA	[90]
LSUB	Reverse, LSU	ACGAACGATTTGCACGTCAG	[88]
*rbc*L-192	Forward, *rbc*L	GGTACTTGGACAACWGTWTGGAC	[91]
*rbc*L-657	Reverse, *rbc*L	GAAACGGTCTCKCCARCGCAT	[91]
TufA-F	Forward, *tuf*A	TGAAACAGAAMAWCGTCATTATGC	[92]
TufA-R	Reverse, *tuf*A	CCTTCNCGAATMGCRAAWCGC	[92]

## Data Availability

The original data presented in the study are openly available in the National Marine Biodiversity Institute of Korea and the Korean Collection for Type Cultures at MABIK LP00000147 and KCTC 15413BP, respectively.

## Supplementary Materials

The following supporting information can be downloaded at: https://www.mdpi.com/article/10.3390/plants13213008/s1, Table S1. Fatty acid percentage comparisons of *A. quadricellulare* strains, other microalgae, and selected second-generation oil sources.
