# Supplementary material for: Taxonomic, Physiological, and Biochemical Characterization of Asterarcys quadricellularis AQYS21 as a Promising Sustainable Feedstock for Biofuels and ω-3 Fatty Acids
Source: Plants (Basel). 2024 Oct 28;13(21):3008. doi: 10.3390/plants13213008 (PMC11548009; doi:10.3390/plants13213008)
Supplement: Supplementary file 1 [file plants-13-03008-s001.zip › plants-3243543-supplementary.pdf]

Supplemental Table

Table S1. Fatty acid percentage comparisons of *A. quadricellulare* strains, other microalgae, and selected second-generation oil sources.

Species	Individual Fatty Acids as Percentages of Total Fatty Acids																												**Refer-ences**
	Saturated fatty acids										Monounsaturated fatty acids						Polyunsaturated fatty acids												
	$C_{11:0}$	$C_{13:0}$	$C_{14:0}$	$C_{15:0}$	$C_{16:0}$	$C_{17:0}$	$C_{18:0}$	$C_{20:0}$	$C_{22:0}$	$C_{24:0}$	$C_{16:1}$ ω9	$C_{16:1}$ ω7	$C_{18:1}$ ω9	$C_{18:1}$ ω7	$C_{20:1}$ ω9	$C_{24:1}$ ω9	$C_{16:2}$ ω4	$C_{16:3}$ ω3	$C_{16:3}$ ω4	$C_{16:4}$ ω3	$C_{18:2}$ ω6	$C_{18:3}$ ω6	$C_{18:3}$ ω4	$C_{18:3}$ ω3	$C_{18:4}$ ω1	$C_{18:4}$ ω3	$C_{20:4}$ ω6	$C_{20:5}$ ω3	
A. quadricellu-laris AQYS21	-	-	-	-	25.5	-	0.87	-	-	-	-	-	13.3	-	-	-	-	-	-	-	5.98	-	-	**54.39**	-	-	-	-	This study
A. quadri-cellularis KNU020	-	-	-	-	15.3	-	-	-	-	-	-	6.8	-	-	-	-	-	-	-	14.8	3.6	-	-	41.2	-	-	-	-	[**1**]
A. quadri-cellularis (China)	-	-	12.31	-	56.24	-	-	-	-	-	-	27.49	-	-	-	-	-	-	-	-	-	-	-	-	-	-	-	-	[**2**]
A. quadri-cellularis (Benha, Egypt)	0.34	1.17	0.87	2.94	17.17	0.95	1	-	-	-	8.97	3.55	16.43	2.43	0.23	0.89	-	-	1.23	10.06	3.68	0.18	0.32	24.32	2.87	-	0.41	-	[**3**]
A. quadri-cellularis (Menou-fia, Egypt)	-	-	-	-	20.54	-	0.59	-	-	-	-	-	54.92	-	-	-	2.22	6.49	-	0.91	13.38	-	-	-	-	0.94	-	-	[**4**]
Chlamydo-monas hedleyi MM0020	-	-	-	-	18.3	-	1.2	-	-	-	-	2.6	-	-	-	-	-	-	-	-	9.8	-	-	16.4	-	-	-	-	[**5**]

Chlorella salina MM0063	-	-	-	-	22.5	-	1	-	-	-	-	0.4	2.2	-	-	-	-	-	-	-	10.6	-	-	-	-	-	-	-	[**6**]
Coelastrum microporum IBL-C119	-	-	-	-	25.66	-	2.91	-	-	-	-	1	44.24	-	-	-	-	-	-	-	8.58	11.12	-	-	-	-	-	-	[**8**]
Dunaliella salina LIMS-PS-1511	-	-	-	-	19.3	-	1.6	-	-	-	-	-	3.7	-	-	-	-	-	-	-	5.6	-	-	31.7	-	-	-	-	[**5**]
Graesiella emersonii GEGS21	-	-	-	-	27.5	-	0.6	-	-	-	-	-	22.2	-	0.3	-	-	-	-	-	26.3	1.0	-	22.1	-	-	-	-	[**9**]
Haemato-coccus la-custris	-	-	-	-	22.49	-	3.15	-	-	-	-	0.64	19.36	-	0.13	-	-	-	-	-	20.23	0.86	-	16.18	-	-	-	0.57	[**10**]
Microglena monadina NFW3	-	-	-	-	25.09	-	0.4	-	-	-	-	3.28	-	-	-	-	-	-	-	-	14.47	-	-	53.01	-	-	-	-	[**11**]
My-chonastes homo-sphaera UTEX2341	-	-	-	-	12.5	-	0.4	-	-	-	-	19.4	4.5	-	-	-	-	-	-	-	2.1	3.6	-	-	-	-	-	31.8	[**7**]
Jaagichlo-rella luteo-viridis MM0014	-	-	-	-	20.7	-	1.4	-	-	-	-	-	7.1	-	-	-	-	-	-	-	35.6	-	-	16.2	-	-	-	-	[**12**]

Tetrades-mus obliquus MM0026	-	-	-	-	18	-	1.3	-	-	-	-	2	16.4	-	-	-	-	-	-	-	5.2	-	-	28.3	-	-	-	-	[5]
Second-generation oil sources																													
Jatropha	-	-	-	-	13.4	-	6.4	-	-	-	-	0.8	36.5	-	0.1	-	-	-	-	-	42.1	-	-	0.2	-	-	-	-	[13]
Karanja	-	-	-	-	7.4	-	3.8	-	-	-	-	-	65.6	-	-	-	-	-	-	-	15.4	4.4	-	-	-	-	-	-	[14]
Mahua	-	-	-	-	21.5	-	19	-	-	-	-	-	39.1	-	-	-	-	-	-	-	19.6	0.16	-	-	-	-	-	-	[15]
Palm	-	-	-	-	47.9	-	4.23	-	-	-	-	0.04	37	-	-	-	-	-	-	-	9.07	0.26	-	-	-	-	-	-	[16]
Rapeseed	-	-	-	-	3.49	-	0.85	-	-	-	-	-	64.4	-	–	-	-	-	-	-	22.3	8.23	-	-	-	-	-	-	[17]

Supplementary Materials References

1. Hong, J.W.; Kim, S.A.; Chang, J.; Yi, J.; Jeong, J.; Kim, S.; Kim, S.H.; Yoon, S.H. Isolation and description of a Korean microalga, *Asterarcys quadricellulare* KNUA020, and analysis of its biotechnological potential. *Algae* **2012**, *27*, 197-203.
2. Ren, H.Y.; Song, X.; Kong, F.; Song, Q.; Ren, N. Q.; Liu, B. F. Lipid production characteristics of a newly isolated microalga *Asterarcys quadricellulare* R-56 as biodiesel feedstock. *Environ. Sci. Pollut. Res. Int.* **2023**, *30*, 48339-48350.
3. Mohamed, S.; Eladel, H.M.; Battah, M.; Ibrahim, Y. Screening of the potentiality of four green microalgae to be used as feedstock for biodiesel and nutraceutical production. *Egypt. J. Phycol.* **2022**, *23*, 1-33.
4. Morsi, H.; Eladel, H.; Maher, A. Coupling nutrient removal and biodiesel production by *Asterarcys quadricellulare* microalga grown in municipal wastewater. *BioEnergy Res*. **2021**, *15*, 193-201.
5. Jo, S.W.; Kang, N.S.; Lee, J.A.; Kim, E.S.; Kim, K.M.; Yoon, M.; Hong J.W.; Yoon, H.S. Characterization of MABIK microalgae with biotechnological potentials. *J. Mar. Biosci. Biotechnol.* **2020**, *12*, 40-49.
6. Kang, N.S.; Lee, J.A.; Jang, H.S.; Kim, K.M.; Lim, E.S.; Yoon, M.; Hong, J.W. First record of a marine microalgal species, *Chlorella gloriosa* (Trebouxiophyceae) isolated from the Dokdo Islands, Korea. Korean *J. Environ. Biol.* **2019**, *37*, 526-534.
7. Vanderploeg, H.A.; Liebig, J.R.; Gluck, A.A. Evaluation of different phytoplankton for supporting development of zebra mussel larvae (*Dreissena polymorpha*): the importance of size and polyunsaturated fatty acid content. *J. Great Lakes Res.* **1996**, *22*, 36-45.
8. Nascimento, I.A.; Marques, S.S.I.; Cabanelas, I.T.D.; Pereira, S.A.; Druzian, J.I.; de Souza, C.O.; Vich, D.V.; de Carvalho, G.C.; Nascimento, M.A. Screening microalgae strains for biodiesel production: Lipid productivity and estimation of fuel quality based on fatty acids profiles as selective criteria. *Bioenergy Res.* **2013**, *6*, 1-13.
9. Kang, N.S.; Cho, K.; An, S.M.; Kim, E.S.; Ki, H.; Lee, C.H.; Choi, G.; Hong, J.W. Taxonomic and biochemical characterization of microalga *Graesiella emersonii* GEGS21 for its potential to become feedstock for biofuels and bioproducts. *Energies* **2022**, *15*, 8725.
10. Damiani, M.C.; Popovich, C.A.; Constenla, D.; Leonardi, P.I. Lipid analysis in *Haematococcus pluvialis* to assess its potential use as a biodiesel feedstock. *Bioresour. Technol.* **2010**, *101*, 3801-3807.
11. Andrew, A.R.; Yong, W.T.L.; Misson, M.; Anton, A.; Chin, G.J.W.L. Selection of tropical microalgae species for mass production based on lipid and fatty acid profiles. *Front. Energy Res.* **2022**, *10*, 912904.
12. Kim, K.M.; Kang, N.S.; Jang, H.S.; Park, J.S.; Jeon, B.H.; Hong, J.W. Characterization of *Heterochlorella luteoviridis* (Trebouxiaceae, Trebouxiophyceae) isolated from the Port of Jeongja in Ulsan, Korea. *J. Mar.Biosci. Biotechnol.* **2017**, *9*, 22-29.
13. Becker, K.; Makkar, H.P.S. *Jatropha curcas*: A potential source for tomorrow's oil and biodiesel. *Lipid Technol*. **2008**, *20*, 104-107.
14. Goembira, F.; Saka, S. Advanced supercritical methyl acetate method for biodiesel production from *Pongamia pinnata* oil. *Renew. Energy* **2015**, *83*, 1245-1249.
15. Saravanan, N.; Nagarajan, G.; Puhan, S. Experimental investigation on a DI diesel engine fuelled with *Madhuca Indica* ester and diesel blend. *Biomass Bioenergy* **2010**, *34*, 838-843.
16. Crabbe, E.; Nolasco-Hipolito, C.; Kobayashi, G.; Sonomoto, K.; Ishizaki, A. Biodiesel production from crude palm oil and evaluation of butanol extraction and fuel properties. *Process Biochem.* **2001**, *37*, 65-71.
17. Ramadhas, A.S.; Muraleedharan, C.; Jayaraj, S. Performance and emission evaluation of a diesel engine fueled with methyl esters of rubber seed oil. *Renew. Energy* **2005**, *30*, 1789-1800.
